# Supplementary material for: Wrinkle force microscopy: a machine learning based approach to predict cell mechanics from images
Source: Commun Biol. 2022 Apr 14;5:361. doi: 10.1038/s42003-022-03288-x (PMC9010416; doi:10.1038/s42003-022-03288-x)
Supplement: Supplementary file 2 — Supplementary information [file 42003_2022_3288_MOESM2_ESM.pdf]

Supplementary information for
"Wrinkle force microscopy: a machine learning based approach to predict cell mechanics from images"

Honghan Li,[1] Daiki Matsunaga,[1] Tsubasa S. Matsui,[1] Hiroki Aosaki,[1] Genki Kinoshita,[1] Koki Inoue,[1] Amin Doostmohammadi,[2,1] and Shinji Deguchi[1]

[1]*Graduate School of Engineering Science, Osaka University, Toyonaka 5608531, Japan*

[2]*Niels Bohr Institute, University of Copenhagen, Blegdamsvej 17, 2100 Copenhagen, Denmark*

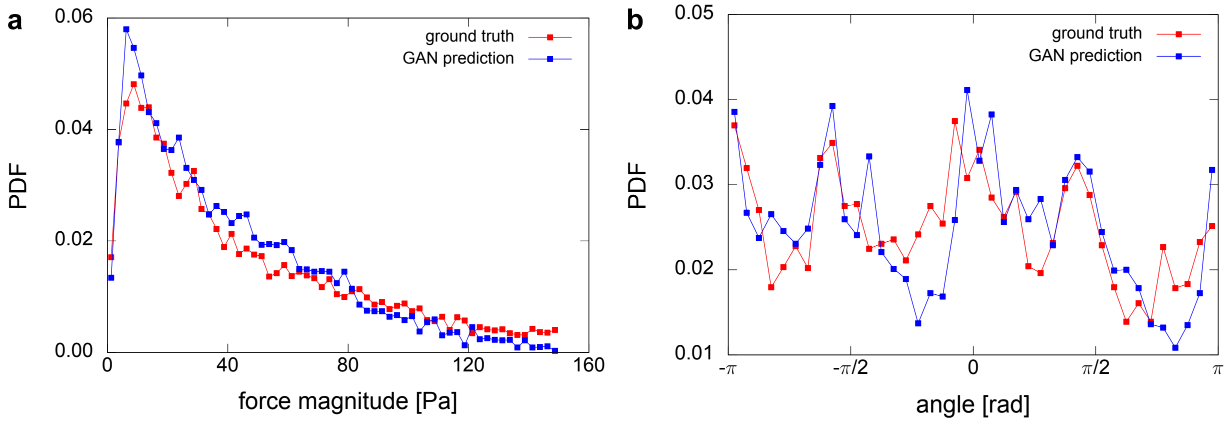

FIG. S1. Histograms of ground truth and GAN predition for (a) force magnitude and (b) force angle. The distributions of GAN prediction have good agreement with that of ground truth.

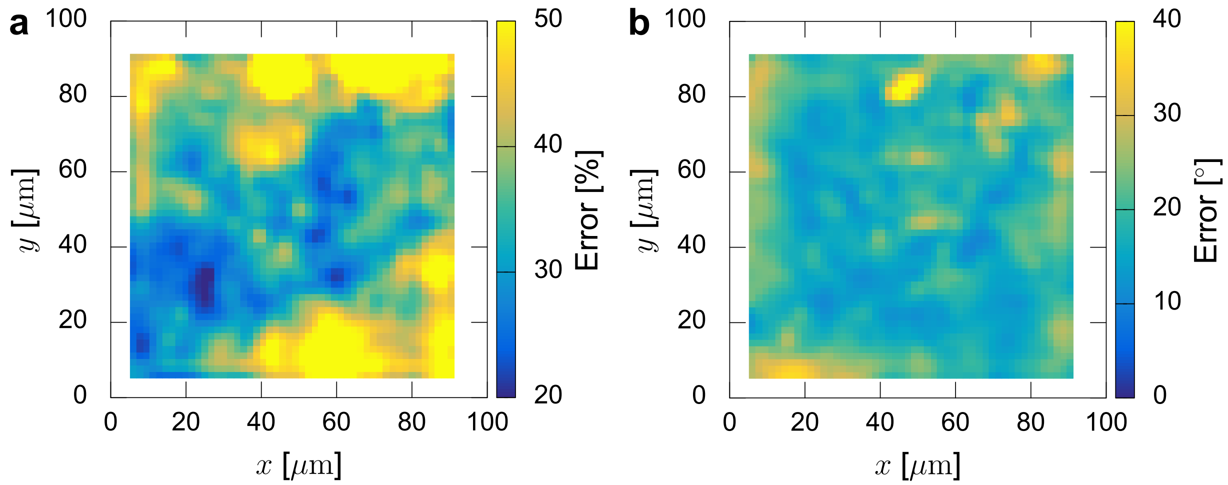

FIG. S2. Spatial error of (a) force magnitude ε_f and (b) force directions ε_θ for 15 test images. Large errors tend to appear at the image edges.

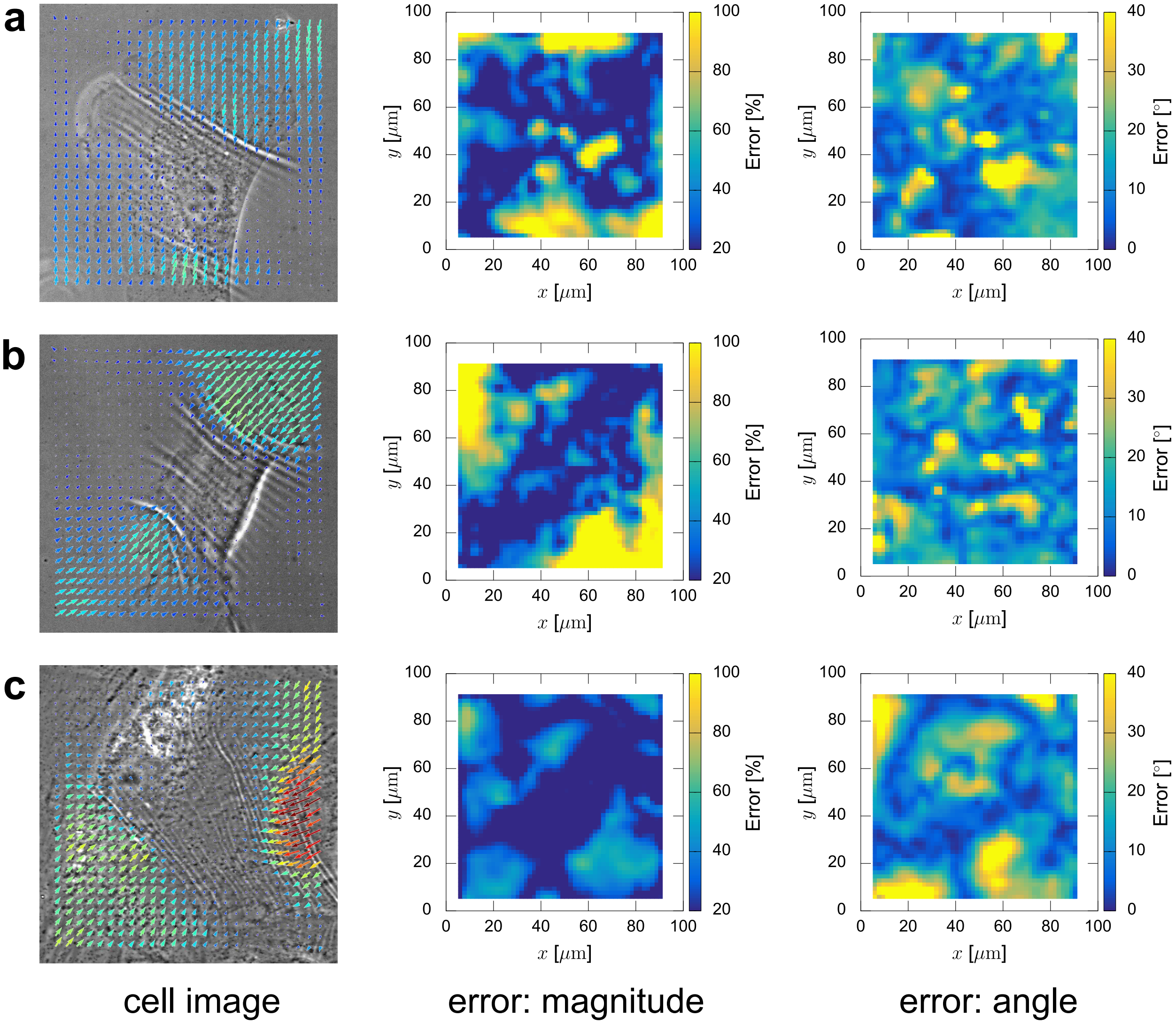

FIG. S3. Spatial error of force magnitude ε_f and force directions ε_θ for 3 cells.

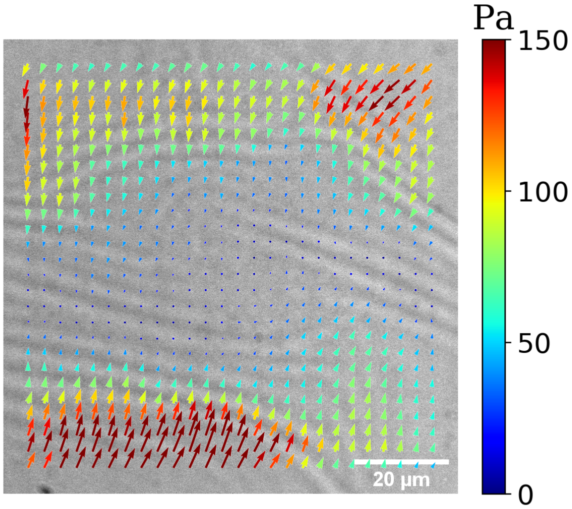

FIG. S4. Distribution of traction force for MEF (Mouse embryonic fibroblast).

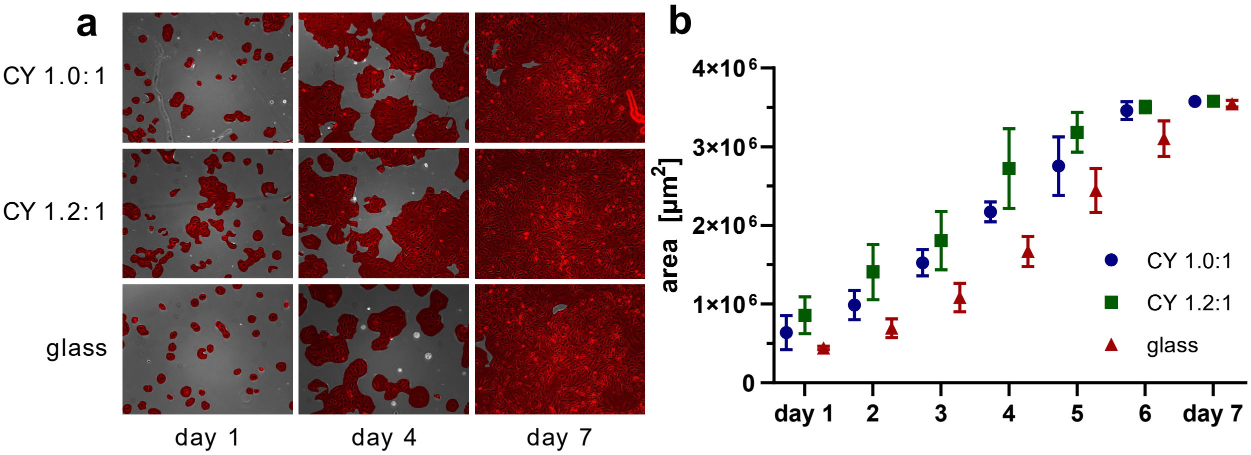

FIG. S5. Cell proliferation for three different substrates: CY with mix ratio 1.2:1.0, CY with 1.0:1 and glass substrates. (a) Cell area (red) for three difference substrates. (b) Time history of the cell area for seven days. Although the growth rate is slower for glass substrates, the qualitative trends are the same. Note that the standard deviations are given from data with 3 different dishes.

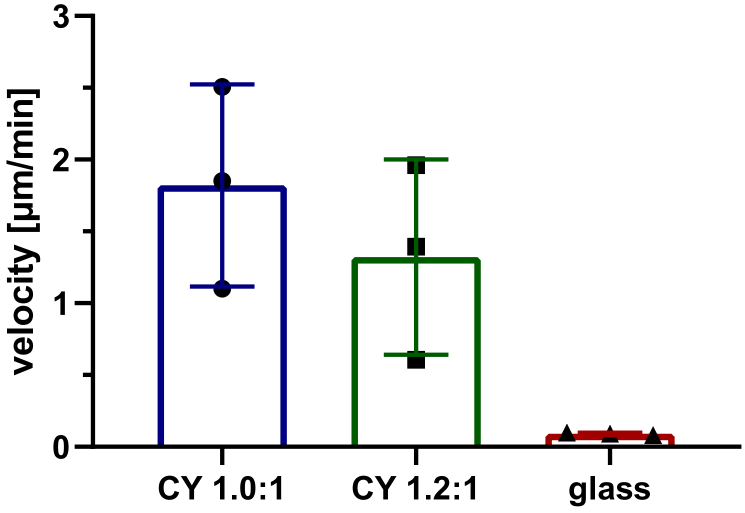

FIG. S6. Cell migration velocities for three different substrates. The standard deviation of each substrate is evaluated with data of 3 different dishes.

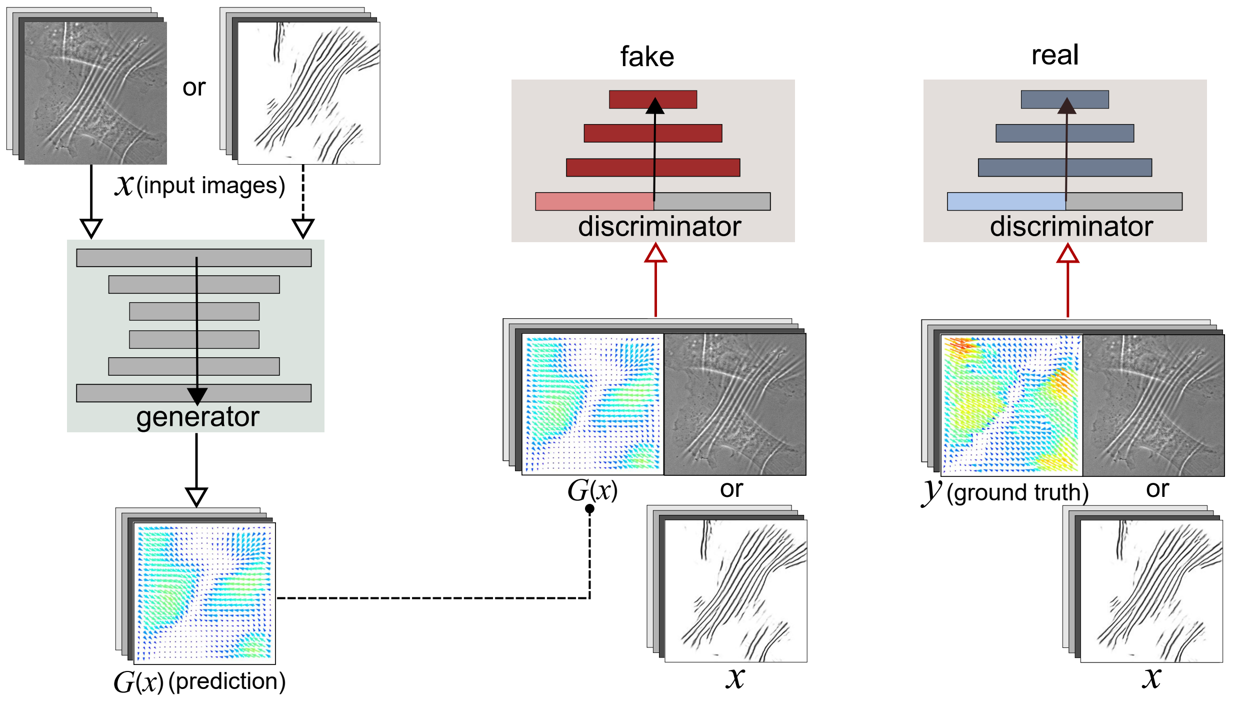

FIG. S7. Schematic showing the structure of GAN (generative adversarial network) utilized in the present work.

SUPPLEMENTAL VIDEO CAPTIONS

- **Movie S1**: Sample of the force estimation using our system. The cell is MEF (mouse embryonic fibroblast). The movie is in 5 minutes/frame. The substrate is prepared by mixing parts A and B of CY 52-276 with a weight ratio of 1.1:1.
- **Movie S2**: Sample of the force estimation using our system. The conditions are same as Movie 1.
- **Movie S3**: Sample of the force estimation using our system. The conditions are same as Movie 1.
- **Movie S4**: Sample of the force estimation using our system. The conditions are same as Movie 1.
