# Supplementary material for: Wrinkle force microscopy: a machine learning based approach to predict cell mechanics from images
Source: Commun Biol. 2022 Apr 14;5:361. doi: 10.1038/s42003-022-03288-x (PMC9010416; doi:10.1038/s42003-022-03288-x)
Supplement: Supplementary file 3 — Description of Additional Supplementary Files [file 42003_2022_3288_MOESM3_ESM.pdf]

Description of Additional Supplementary Files

File name: Supplementary Data 1
Description: Source data for the graphs and charts in the figures.

File name: Movie S1
Description: Sample of the force estimation using our system. The cell is MEF (mouse embryonic fibroblast) The movie is in 5 minutes/frame. The substrate is prepared by mixing parts A and B of CY 52-276 with a weight ratio of 1.1:1.

File name: : Movie S2
Description: Sample of the force estimation using our system. The conditions are same as Movie 1.

File name: : Movie S3
Description: Sample of the force estimation using our system. The conditions are same as Movie 1.

File name: : Movie S4
Description: Sample of the force estimation using our system. The conditions are same as Movie 1.
